# Low-Engine-Order Forced Response Analysis of a Turbine Stage with Damaged Stator Vane

**DOI:** 10.3390/e26010004

**Published:** 2023-12-19

**Authors:** Yun Zheng, Xiubo Jin, Hui Yang

**Affiliations:** School of Energy and Power Engineering, Beihang University, Beijing 100191, China; zheng_yun@buaa.edu.cn (Y.Z.); jxbuaa@buaa.edu.cn (X.J.)

**Keywords:** damaged stator vane, low-engine-order (LEO) forced response, aeroelastic simulations, wake and potential field, secondary vortex

## Abstract

A damaged stator vane can disrupt the circumferential symmetry of the design flow for turbine assemblies, which can lead to a low-engine-order (LEO) forced response of rotor blades. To help engineers be able to better address sudden vane damage failures, this paper conducts a mechanism analysis of the LEO forced response of rotor blades induced by a single damaged vane using an in-house computational fluid dynamic code (Hybrid Grid Aeroelasticity Environment). Firstly, it is found that the damaged vane introduces a family of LEO aerodynamic excitations with high amplitudes by full-annulus unsteady aeroelastic simulations of a single-stage turbine. In particular, the LEO forced response of the rotor blades excited by 3EO is 2.01 times higher than the resonance response excited by vane passing frequency, and the LEO resonance risk of the rotor blades is greatly increased. Then, by analyzing the flow characteristics of the wake and potential field of the stator row with a damaged vane, the localized high transient pressure in the notch cavity and the radial redistribution of the secondary vortex at the stator exit are the main sources of the low-order harmonic components in the flow field. Importantly, the interaction mechanisms in two regions with high LEO excitation amplitude on the rotor blade surface are revealed separately. Finally, an evaluation and comparison of a single damaged vane in terms of aerodynamic performance and LEO forced response was carried out. The results of this paper provide a good theoretical basis for engineers to effectively control the resonance response of rotor blades caused by a damaged stator vane in turbine design.

## 1. Introduction

The low-engine-order (LEO) forced response is forced vibration driven by low-order harmonic external forces, where the fundamental blade modes of the rotor blades at low nodal diameters (ND) are excited with higher vibration levels [1,2,3]. This increases the risk of high cycle fatigue failure of the rotor blades considerably, endangering the safe operation of aero engines. Also, it has been shown that any loss of symmetry in the flow field may cause an LEO forced response [1,4,5,6]. However, the stator vanes of the turbine, as high-temperature components downstream of the combustion chamber, experience creep, fatigue, oxidation and corrosion, and sudden damage failures at the trailing edges occur regularly [7,8,9]. Geometric changes in the stator vanes not only affect the work of the stator row but may also destroy the circumferential symmetry of the design flow and cause the LEO forced response on rotor blades. Currently, such sudden failures are often unpredictable and the exact excitation mechanism that causes LEO forced response is not known. Therefore, conducting studies on LEO forced response induced by damaged stator vane is crucial in the design and operation of aero-engines.

Many studies have been carried out on the geometric variation of stator vanes [10,11,12]. Most of these studies have addressed the variation of common geometrical design parameters such as throat width [1,13] and stagger angle [14,15,16], blade count ratio [17,18], etc., and some studies deal with small wear of the blade geometry [19,20]. Previous literature on the forced response induced by damaged stator vanes, on the other hand, is limited. Carleton University first [21,22,23,24] studied a single turbine vane damaged at the mid-span of the trailing edge. Experimental and numerical simulations showed that a swept leakage from the pressure surface to the suction surface like the tip leakage flow is formed at the edge of the notch. Two counter-rotating vortices appear to be generated downstream of the notch. However, the study did not provide a visualization flow field to confirm this finding, nor did it consider the possible LEO forced response due to a single damaged stator vane. Mare et al. [25] evaluated for the first time the LEO forced response on rotor blades by a stator row containing one severely damaged vane through numerical simulations. It is found that large flow separation occurs in the notch cavity on the suction side of the damaged vane, and the amplitude of LEO modal forces for the damaged case was eight times larger than that of the undamaged case. However, the throat width of the undamaged original stator row was not uniform, which may made it difficult to analyze the excitation sources. It can only be concluded qualitatively that the large radial extent of flow disruption on the rotor blade is the main source of excitation for the low-order modes with high response levels. Meyer et al. [26] found that large damage to the stator vane will lead to the development of two strong vortices by three-dimensional flow field visualization. This strong distortion is limited to the neighboring passages of the damaged vane. It is concluded that the distortion of the flow field at the stator exit causes periodic changes in the loading of the rotor blades and becomes the main cause of exciting the low-order modes. However, a stator assembly with a large number of vanes with different damage sizes randomly placed over the circumference was chosen for the study, and the fundamental LEO excitation components that would be introduced by damaged vanes are difficult to identify. The knowledge of the LEO excitation components is a prerequisite for the identification of LEO resonance risks. So, the study on the forced response for a single damaged vane may be more helpful in revealing the underlying mechanism of the LEO forced response of the rotor blade excited by the damaged stator. Overall, the damage levels of stator vanes that have been studied in the previous literature are very large. The damage ranges from approximately 40% chord or greater and the circumferential distribution of multiple damaged vanes in the stator row is also complicated. Such severe damage is rare and extreme during early engine operation. Damaged vanes with smaller trailing edge notches may appear earlier, and it is important to assess whether such small perturbations still result in LEO forced responses at high levels. Furthermore, the main aerodynamic excitation sources in the turbine stage include wakes and potential fields [27,28]. Controlling the strength of the wake, potential field and their interactions may control the level of aerodynamic excitation on rotor blades and thus blade response. These aerodynamic measures to control blade response in terms of unsteady aerodynamics have emerged as possibilities for controlling the vibrational behavior of the rotor blades [29,30,31], which in turn requires a deeper understanding of the unsteady flow. Therefore, one of the aims of this paper is to determine the relative influence of the wake and potential field of a single damaged stator on the LEO excitation of rotor blades from the aspect of unsteady aerodynamics. This will help engineers both to take constructive measures in turbine design and to make wiser decisions in response to sudden failures during engine operation.

In this paper, a full-annular unsteady aeroelasticity simulation of a single-stage turbine is performed using an in-house CFD code, Hybrid Grid Aeroelasticity Environment (HGAE), and underlying mechanism of the LEO excitation associated with a single damaged vane is investigated in depth. By analyzing the flow characteristics of the wake and potential field at the stator row with a single damaged vane, the main sources of the low-order harmonic components at the stator exit (i.e., the low-order harmonic external forces that cause the LEO’s forced response) are identified for the first time. The interaction mechanism of the low-order harmonic components of the flow field on different locations of the blade is discovered by combining the time–space diagram and the transient flow field. In addition, this paper adds the evaluation and comparison of a single damaged vane in terms of aerodynamic performance and LEO forced response. This work is also one of the first studies to provide a detailed description of the flow physics associated with the increased LEO resonance risk induced by a single damaged vane using a full-annular unsteady aeroelasticity environment.

## 2. Geometrical Model and Numerical Methods

### 2.1. Single-Stage Turbine

The baseline case employed in this study is a single-stage turbine, which consists of a stator and rotor. The stator row has 16 vanes with 75 mm height and 0.77 aspect ratio. The rotor row has 47 blades, each rotor blade has 74 mm height and 1 mm tip clearance, which is about 1.35% of the outlet blade height. The stator was modeled with zero clearance. More design parameters of the geometrical model are shown in Table 1.

### 2.2. Numerical Methods

The study in this paper is realized by employing the in-house CFD code HGAE, which is used to simulate unsteady flow and aeroelasticity in three dimensions. HGAE has been validated in various aerodynamic and aeroelastic cases [31,32,33,34] for close to two decades and is suitable to be applied to the solutions of complex flows in turbomachinery. More details of the in-house code can be found at Zheng [35,36].

#### 2.2.1. Aerodynamic Models

The unsteady compressible Navier–Stokes equations for a control body Ω with boundary ∂Ω can be expressed in the integral form shown below:

(1) $$\frac{\partial }{\partial t}\int_{\Omega }\vec{U}d\Omega +\oint_{\partial \Omega }\left(\vec{F_{c}}-\vec{F_{v}}\right)dA=\int_{\Omega }\vec{H}d\Omega ,$$

where the surface area of the element is denoted using dA . Equation (1) also contains the vector of conservative variables ( $\vec{U}$ ), the convective ( $\vec{F_{c}}$ ) and viscous flux vectors ( $\vec{F_{v}}$ ), and the source term vector ( $\vec{H}$ ). They can be expressed in the following equations:

(2) $$\vec{U}=\left[\begin{array}{c} \rho \\ \rho u\\ \rho v\\ \rho w\\ \rho E \end{array}\right],$$

(3) $$\vec{F_{c}}=\left[\begin{array}{c} \rho V\\ \rho uV+n_{x}p\\ \rho vV+n_{y}p\\ \rho wV+n_{z}p\\ \rho HV \end{array}\right]+V_{t}\left[\begin{array}{c} \rho \\ \rho u\\ \rho v\\ \rho w\\ \rho E \end{array}\right],$$

(4) $$\vec{F_{v}}=\left[\begin{array}{c} 0\\ n_{x}\tau_{xx}+n_{y}\tau_{xy}+n_{z}\tau_{xz}\\ n_{x}\tau_{yx}+n_{y}\tau_{yy}+n_{z}\tau_{yz}\\ n_{x}\tau_{zx}+n_{y}\tau_{zy}+n_{z}\tau_{zz}\\ n_{x}\Theta_{x}+n_{y}\Theta_{y}+n_{z}\Theta_{z} \end{array}\right],\;\vec{H}=\left[\begin{array}{c} 0\\ \rho f_{e,x}\\ \rho f_{e,y}\\ \rho f_{e,z}\\ \rho {\vec{f}}_{e}\cdot \vec{v}+{\dot{q}}_{h} \end{array}\right],$$

The governing equations for the multi-block grid were discretized using the finite volume method. The convective terms and central differences for the diffusive fluxes were calculated using Roe’s upwind scheme and the Monotone Upwind Scheme for Conservation Law extrapolation [37,38]. The numerical scheme achieves second-order accuracy. Jameson’s dual time-stepping method with 15 sub-iterations was employed to improve the accuracy of the time-advancing solution for the unsteady calculations [39]. A two-equation Shear Stress Transport model is chosen to solve the Reynolds-Averaged Navier–Stokes (RANS) equations [40]. The turbulent intensity at the inlet was 5%.

#### 2.2.2. Structural Models

For the structural model, the structural dynamics equations of the linear aeroelastic model are employed:

(5) $$M\ddot{x}+C\dot{x}+Kx=p\left(t\right)An,$$

where the mass matrix M , stiffness matrix C , damping matrix K, and displacement vector x are on the left side of the equation, which can be obtained by finite element calculations. The right side of the equation is the product of the pressure vector p(t) , the application area ( A ), and the normal unit vector ( n ), which represents the aerodynamic vector on the blade surface. The modalized structural dynamics equations can be obtained by coordinate transformation x=φq :

(6) $$\ddot{q_{i}}+\left(2\zeta_{i}\omega_{i}\right)\dot{q_{i}}+\left({\omega_{i}}^{2}\right)q_{i}={\varphi_{i}}^{T}p\left(t\right)An=\Theta_{i}\left(t\right)\;\;\;\;\;\;\;\;\;\;\left(i=1,N\right),$$

where $\varphi_{i}$ is the mode shape vector and $q_{i}$ represents the modal deflection. The modal damping and natural frequency of mode i are denoted by $\zeta_{i}$ and $\omega_{i}$ , respectively. $\Theta_{i}\left(t\right)$ is the modal projection of the aerodynamic load vector p(t)An on the mode shape $\varphi_{i}$ . N represents the number of mode shapes to be analyzed.

The in-house code HGAE integrates the flow solver and the structural solver described above and can be run either coupled or uncoupled when conducting numerical simulations of the forced response. The method selection is based on the blade size and the actual vibration amplitude [41,42,43]. Compared with fan blades with large aspect ratios, the rotor blades of the turbine stage in this paper usually have smaller amplitude vibrations, so the selection of the uncoupled approach is appropriate [31,44]. In the uncoupled solver, the blade motion will not be considered. During preprocessing, HGAE interpolates the mode shapes of the rotor blades onto the fluid mesh. The data exchange between the structural and fluid boundaries (e.g., transient pressures) takes place at each time step during the uncoupled computation. The unsteady pressures on the blades are all converted to modal forces (Equation (6)) by HGAE.

(7) $$\Theta_{i}\left(t\right)=\sum_{j=1}^{nodes}\varphi_{ij}\left(\Delta A_{j}n_{j}\right)p_{j}\left(t\right),$$

where j is the node index. The strength of the modal forces for a particular mode is determined by the pressure perturbation and the correlation between the pressure perturbation and the structural mode shape. The actual time histories of the modal forces will be output when the uncoupled solver calculations are completed. The response strength of the rotor blade can be obtained by post-processing the modal forces.

## 3. Grid Independence and Code Verification

### 3.1. Grid Independence

**Figure 1:**
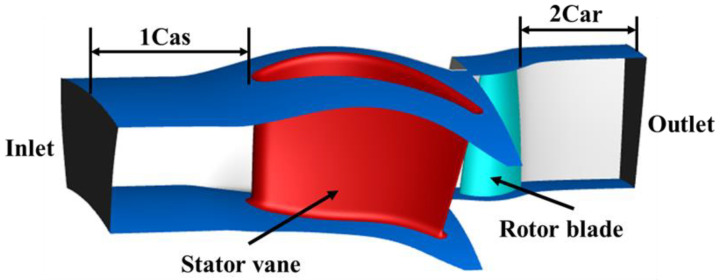
Computational domain of the turbine stage.

Figure 1 illustrates the computational domain of the turbine stage. The inlet boundary is 1 Cas (stator chord) away from the leading edge of the stator vanes, and the inlet total pressure and total temperature are 510,000 Pa and 1480 K. The static pressure (237,000 Pa) is applied to the hub (radial pressure equilibrium) at the outlet boundary, which is 2 Car (rotor chord) away from the trailing edge of the rotor blades, in order to ensure the non-reflecting boundary condition.

**Figure 2:**
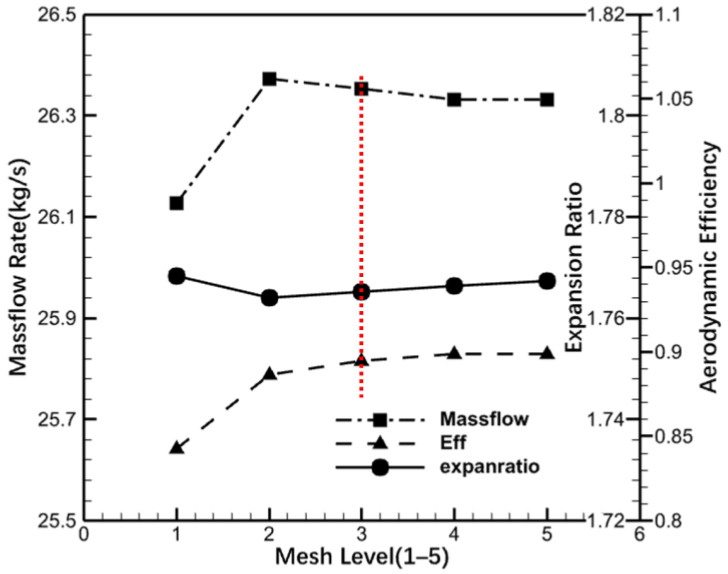
Grid independence analysis.

The computational domain of the turbine stage was meshed using structured hexahedral elements and grid independence was achieved by considering five different grid configurations. All the solutions in the grid-independence study were single-passage steady solutions at the design operating conditions (Table 1). A mixing plane was used for the interfaces between the stator and the rotor rows, and periodic boundary conditions were defined for the stator and rotor rows. The aerodynamic performance of the turbine stage with different grid configurations was evaluated in terms of static pressure distribution on the blade surface, exit flow angle, expansion ratio, aerodynamic efficiency, and mass flow rate, respectively. In this paper, only the results on mass flow rate, aerodynamic efficiency, and expansion ratio are shown in Figure 2.

Based on the considerations of accuracy and computational cost, the Level 3 grid configuration was selected for subsequent analysis in this study. The chosen grid has 1,084,020 total nodes in the single-passage turbine stage, in which the stator and rotor rows employ 69 × 85 × 37 and 61 × 101 × 25 grid distributions in the streamwise, spanwise, and pitchwise directions, respectively. The number of mesh points in the two blade rows is 590,835 and 493,185, respectively. The near-wall grid resolution with y^+^ < 1 satisfies the requirements of the SST k-w turbulence model.

### 3.2. CFD Code Verification

CFD code verification is carried out by comparing the simulation results between the in-house code and the commercial software (NUMECA 17.1). Due to the aerodynamic excitation (wake and potential field, etc.), and aerodynamic performance that are the focus of this paper, the accuracy of the HGAE is confirmed in terms of the static pressure distribution on the blade surface, the exit secondary flow, the pressure field at the S1 flow surface, and the aerodynamic performance of the turbine stage, respectively. The results of both CFD codes are in good agreement. In this section, only the results of aerodynamic efficiency and expansion ratio are shown (Table 2). Among them, the aerodynamic efficiency is defined as follows:

(8) $$\eta =\frac{h_{t\;in}-h_{t\;out}}{h_{t\;in}-h_{t\;is\;out}},$$

where $h_{t\;in}$ and $h_{t\;out}$ are the total inlet enthalpy and total outlet enthalpy, and $h_{t\;is\;out}$ is the isentropic total enthalpy at the outlet.

## 4. Damaged Stator Vane Case

**Figure 3:**
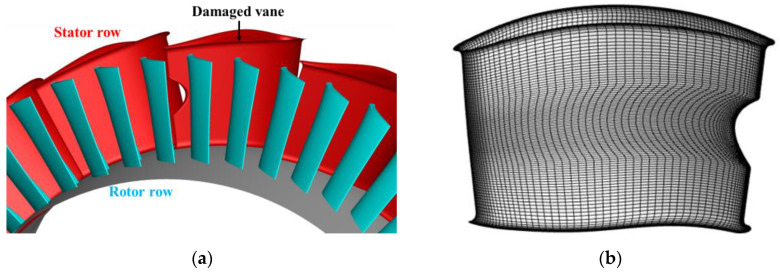
Diagram of damaged vane. (**a**) Damaged vane in the turbine stage; (**b**) Mesh for damaged vane.

As mentioned above, smaller trailing edge notches may generally occur earlier, and the surface temperature distribution of the turbine vane also typically shows high temperatures in the midspan of the trailing edge [23,24]. In this study, a curved trailing edge notch with a maximum damage depth of 12% chord length was used, and the damage in the spanwise direction ranged from 34–69% blade height (Figure 3a). Damaged vane geometry was modeled in UG by cutting the original vane with a three-dimensional (3D) cone. Compared with the undamaged vane, the mesh of the damaged vane was locally encrypted to identify the unsteady flow features near the notch (Figure 3b).

Furthermore, in this paper, a turbine stage case without a damaged stator vane is noted as Baseline case, and Damage case represents a turbine stage case with a damaged stator vane.

## 5. Rotor Campbell Diagram

Campbell diagrams are a common method to identify the operating conditions of rotor blade resonance response. The excitation frequency lines are plotted at a fixed slope determined by the rotational frequency (and its multiples), which represent the frequency of each engine order (EO). The natural frequency lines are obtained by using the finite element method to perform a modal analysis of the rotor blade at different speeds.

**Figure 4:**
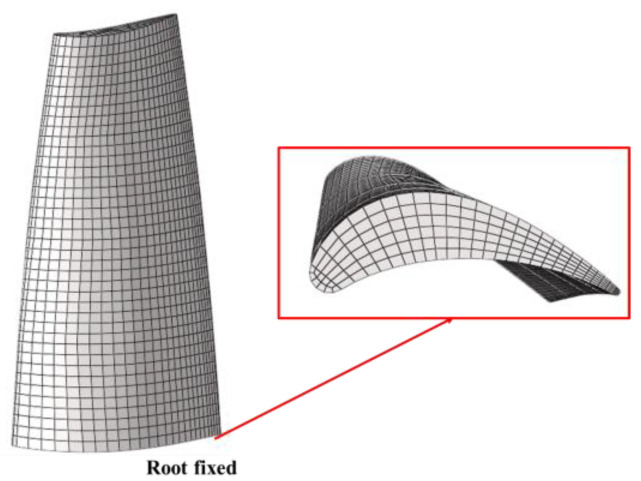
Finite element model of the rotor.

**Figure 5:**
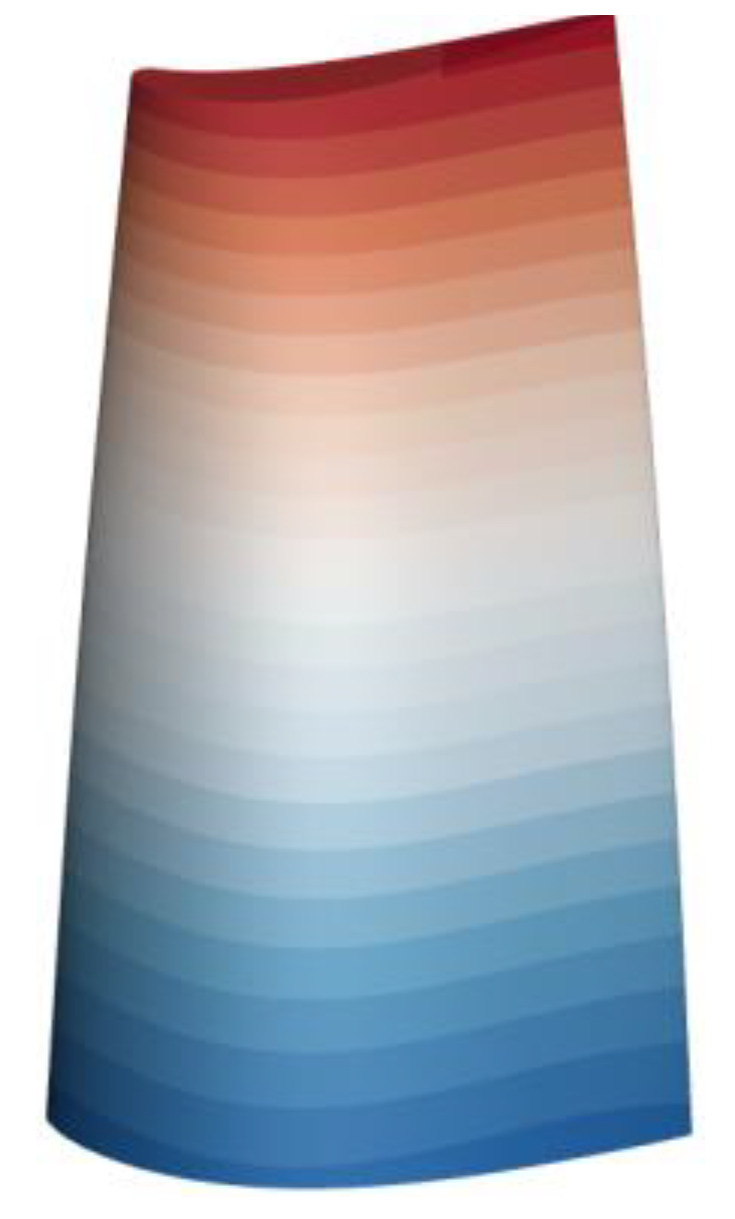
First modal shape (M1).

**Figure 6:**
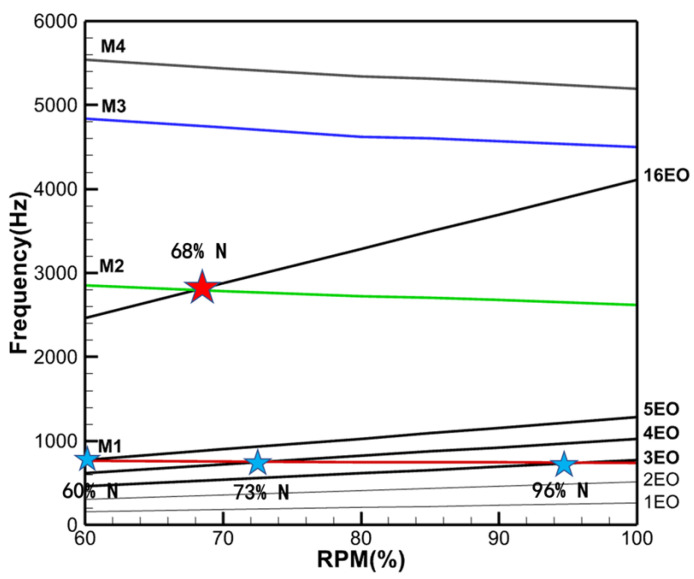
The Campbell diagram of the rotor blade.

The finite element model of the rotor was established using hexahedral elements by only applying fixed constraints to the blade root (Figure 4). The selected blade material has 155 GPa elastic modulus, 0.31 Poisson’s ratio, and 8.44 g/cm^3^ density. The first modal shape obtained from modal analysis in ANSYS are shown in Figure 5. The Campbell diagram of the turbine stage is presented in Figure 6, where the first four orders of natural frequency lines are given.

The rotor blade is excited by the upstream wake and potential field, so the dominant vane passing frequency (VPF) in the flow field is 16 (number of stator vanes) times the rotational frequency, which corresponds to the 16EO excitation line in the Campbell diagram. The 16EO excitation line will excite the 16 node diameter (ND) response of the rotor blade. In the Campbell diagram, the 16EO excitation line intersects the second-order mode (M2/16ND) (solid green line) at 68% $n_{design}$ (red star). This indicates that the stator VPF will induce the M2/16ND resonance, which is the rotational speed at which the preliminary unsteady CFD calculations are carried out. Meanwhile, it should also be noted that there also exists an intersection of the 3–5 EO excitation line with the first-order mode (M1) (red solid line) in the range of 60–100% $n_{design}$ (blue stars). If external aerodynamic excitation at these orders exists, LEO resonance of the rotor blade can be induced, and it becomes particularly important to assess the resonance risk, which is the main subject of this study.

## 6. Results

### 6.1. Identification of LEO Excitation Components

**Figure 7:**
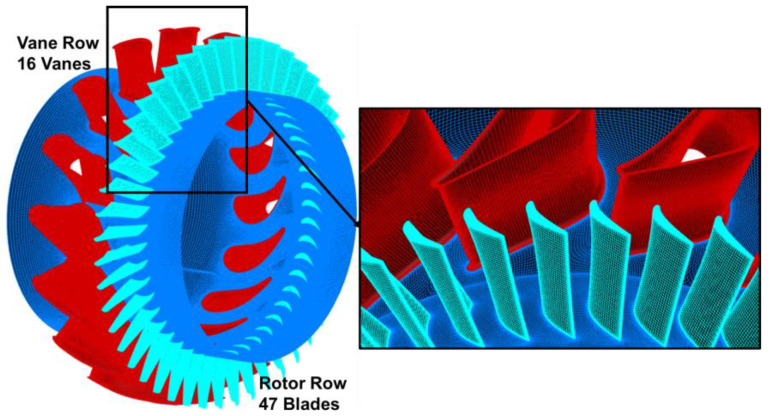
Full annular mesh of Baseline case.

The first major step in assessing the level of LEO forced response induced by the damaged stator row is to identify the LEO aerodynamic excitation components in the flow field. Then, a subsequent study is conducted by determining the rotational speed for the LEO resonance risk through Figure 6. Therefore, the preliminary numerical investigations are full-annulus unsteady calculations using HGAE for the Baseline case and the Damage case at 16EO crossing speed (68% $n_{design}$ ). The full annular mesh is obtained by rotationally duplicating the single passage mesh, and the number of full annular mesh points for the turbine stage is about 32 million. The sliding plane is employed to allow the wake disturbances and potential field disturbances of the stator row to propagate to the rotor row, and Figure 7 shows the full annular mesh of the Baseline case.

The converged solution of the steady simulation for the full annular flow field of the turbine stage is used as the initial flow field for the unsteady calculations. The unsteady calculation employs Jameson’s dual time-stepping. Considering the calculation accuracy and time cost, the time step of the unsteady calculation is set to be $3.787\times 10^{-6}\;s$ . It takes 32-time steps for the rotor blades to turn through 1 rotor pitch and about 94 steps to turn through 1 stator pitch. There are two main criteria for the selection of the initial iteration steps, the first is that the dominant aerodynamic parameters (inlet and outlet flow rates, efficiency, axial force, etc.), show significant time periodicity with the time step iteration, and the second is that the dominant aerodynamic parameter decreases by at least $10^{-3}$ within the 15 sub-iteration steps. Considering that the unsteady flow field of the turbine stage mostly needs to be post-processed on the computational data of the entire cycle, the total iteration step will be one rotation cycle more than the initial iteration step.

**Figure 8:**
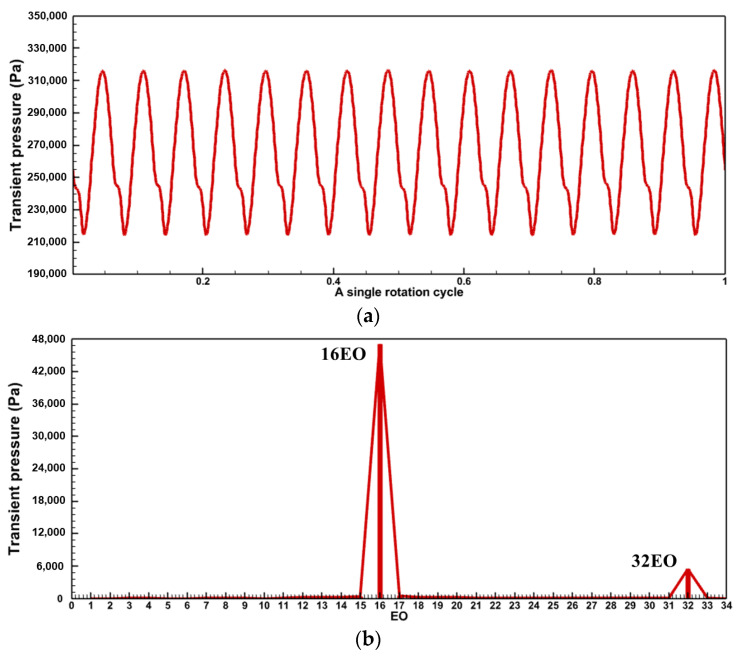
Results on the transient pressure for Baseline case. (**a**) Time domain; (**b**) Frequency domain.

One of the primary techniques for analyzing the frequency components of the aerodynamic excitation in a flow field is to perform a Fourier transform of the unsteady pressure. The preliminary analysis of the excitation components was carried out in Baseline case. The time and frequency domains of the transient pressure during a single rotation cycle at a monitoring point of the rotor blade are shown in Figure 8. The rotor blades are subjected to the stator wake and potential field, and the transient pressure profile shows pressure pulsations of equal amplitude with 16 periods (Figure 8a). The main harmonic (Figure 8b) in the frequency domain results also appears at 16EO, which is the VPF. And the amplitude of the second-order harmonic (32EO) decreases to 1/7 of the amplitude at 16EO. There are no low-order aerodynamic excitations in the Baseline case, except for the frequency components associated with the stator VPF.

**Figure 9:**
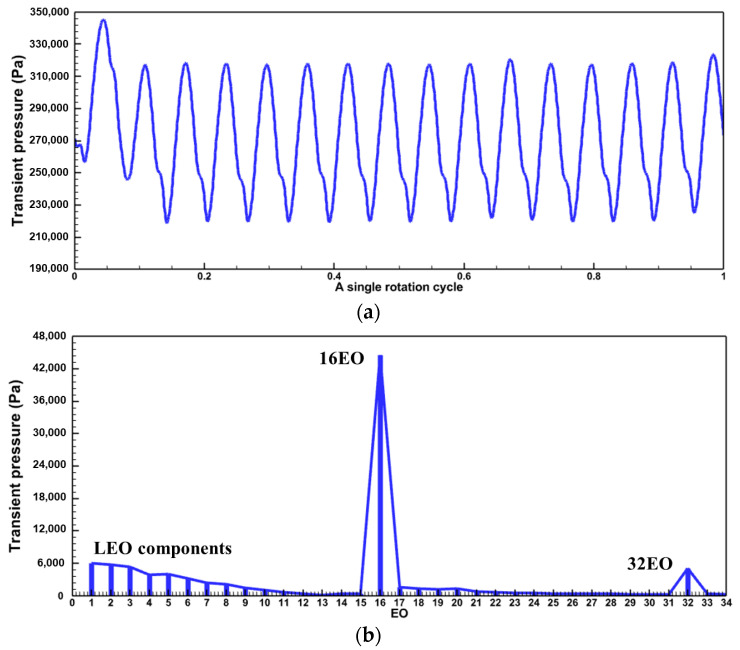
Results on the transient pressure for Damage case. (**a**) Time domain; (**b**) Frequency domain.

For the Damage case where a damaged blade exists in the stator row, the time domain profile of the transient pressure during a single rotation period (Figure 9a) exhibits a single region of high transient amplitude over 16 pulsating periods. The frequency spectrum plot (Figure 9b) exhibits much more noteworthy excitation components. 

In addition to the VPF and its multiples consistent with those in the Baseline case (Figure 8), multiple LEO excitation components can be found. The amplitudes of 1-3 EO are high, about 1/7 of the amplitude of the VPF (16 EO), which is even greater than or equal to the amplitude of the 2VPF (32 EO). As can be seen in Figure 6, a single damaged vane produces three resonance risks to the turbine stage on the 1st mode (M1) in the 60–100% $n_{design}$ : 3EO crossing speed (96% $n_{design}$ ), 4EO crossing speed (73% $n_{design}$ ), 5EO crossing speed (60% $n_{design}$ ). Whether a single damaged blade presents a potential danger to the safe operation of the turbine stage needs to be determined by calculating the resonance response at each resonance rotational speed.

### 6.2. Aeroelastic Simulation for Evaluating Response Levels

Preliminary unsteady calculations show important findings that there are three LEO resonance risks in the turbine stage when a single vane is damaged. The Campbell diagram can only determine the operating speeds at which resonance is likely to occur; it cannot obtain the response intensity at the resonant speeds. Meanwhile, it is difficult to avoid all the resonance conditions in the Campbell diagram in engineering applications, especially the risk of sudden LEO resonance in the 60–100% $n_{design}$ range in this study. Therefore, it is very important to conduct further aeroelastic calculations using HGAE to evaluate the response level of the three resonance conditions.

Similar to the setup of the unsteady simulation, the initial flow field for the aeroelasticity calculations at each resonance condition uses the converged solutions obtained in the steady simulation of the full annular flow field. To ensure the unsteady simulation accuracy for different resonance conditions, it is still set as the standard of unsteady time step that “it takes 32-time steps for the rotor blade to turn through 1 rotor pitch, and 94-time steps to turn through 1 stator pitch”. The unsteady time steps for the three LEO resonance conditions, 3EO crossing, 4EO crossing, and 5EO crossing, were set to be $2.672\times 10^{-6}\;s$ , $3.549\times 10^{-6}\;s$ , and $4.317\times 10^{-6}\;s$ , respectively. 

In addition, in order to better reflect the LEO resonance intensity, aeroelastic calculations were also performed for the 16EO crossing of Baseline case. The unsteady time step is set to $3.787\times 10^{-6\;}s$ , and the response level in this case is considered as the baseline response. The total iteration steps for the aeroelastic calculations were set in the same way as in the case of the unsteady aerodynamic calculations.

Assessing the resonance response level can generally be quantified by calculating the maximum vibration amplitude, which can usually be calculated using the generalized blade deflection equation [42,43,44]:

(9) $$X_{max}=\frac{\Theta Q\Phi_{max}}{{\omega_{r}}^{2}},$$

where Θ is the amplitude of the modal force at the relevant frequency after obtaining the periodic solution; the value of the maximum modal shape obtained from the modal analysis is denoted as $\Phi_{max}$ ; and the Q  factor, which represents the aerodynamic damping (structural damping is not considered in this paper), is defined as follows:

(10) $$Q=\frac{1}{2\zeta },$$

where the aerodynamic damping ratio ζ can be obtained by flutter analysis (coupled approach).

The response of each case is normalized by the maximum vibration amplitude $X_{max,baseline}$ of the 16EO crossing in the Baseline case. The normalized maximum vibration amplitude $X_{max,nor}$ is defined as follows:

(11) $$X_{max,nor}=\frac{X_{max}}{X_{max,baseline}}.$$

The normalized response $X_{max,nor}$ for each LEO resonance condition is given in Table 3. The results show that the response of the 3EO crossing is 2.01 times that of the baseline response excited by the VPF. The responses of the 4EO crossing and the 5EO crossing also reach 1.797 and 1.804 times the baseline response, respectively.

Aeroelastic calculations for resonance conditions demonstrate that an individual damaged vane induces the LEO resonance response of the rotor blade. The response level is much higher than the intensity of the resonance response excited by the VPF, which seriously jeopardizes the safe operation of the turbine stage. To detect the failure of individual damaged vane earlier in engine operation and to better avoid or take measures to mitigate the possible LEO resonance response problem, it is imperative to explore the physical mechanism of LEO forced response of rotor blades induced by a single damaged vane. This study will also provide ideas for troubleshooting after engine failures occur.

### 6.3. Discussion on Flow Mechanisms

Differing from self-excited vibration, the main characteristic of forced response is the presence of external excitation, and the LEO forced response induced by a single damaged vane is caused by the upstream unsteady flow (wake and potential field). A better understanding of the unsteady flow helps to develop the aerodynamic measures that control aerodynamic excitations and hence blade responses. Therefore, the identification of the wake and potential field and their interactions is key to understand the underlying mechanism. The next section focuses mainly on the unsteady flow in the turbine stage to assess and understand the aerodynamic excitation mechanism that induces the LEO forced response.

**Figure 10:**
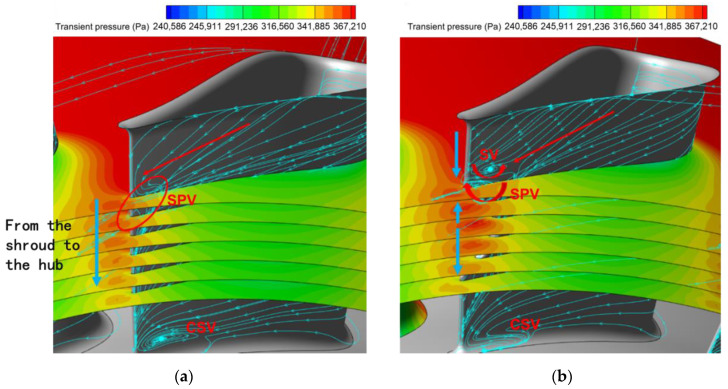
Exits for different stator vanes. (**a**) The undamaged vane; (**b**) The damaged vane.

The flow fields around the undamaged and damaged vanes are shown in Figure 10, respectively. The notch of the damaged vanes is located from 34% to 69% span. Therefore, multiple S1 planes were intercepted near the mid-span along the spanwise direction to characterize the flow at the stator vane passage and outlet. Transient pressure contour plots were applied to these S1 planes. A blade stack design that considers radial pressure equilibrium results in a gradual increase in transient pressure at the trailing edge of the stator outlet from the hub to the shroud (root to tip). This pressure gradient across the spanwise direction is represented as the blue arrow in Figure 10a. The limiting streamlines on the suction side of the blades and the 3D streamlines near the trailing edge are then used to identify the secondary flow characteristics of the aft passage in the stator row.

The limiting streamlines of the undamaged vane surface can be seen as the shroud passage vortex (SPV) and the corner separating vortex (CSV) at the hub. The SPV is formed at the front of the passage, and it keeps on entraining the low-energy fluids outside of the boundary layer on the shroud and vane suction surfaces during its propagation downstream. It eventually flows out of the stator passage in the direction of the red arrow shown in Figure 10a, influenced by the radial pressure gradient. The limiting streamline of the vane surface curving upstream in the red circle in Figure 10a is the trace of the SPV flowing out at the trailing edge of the stator vane.

The flow characteristics of the trailing edge for the damaged vane (Figure 10b) are different from the studies of previous literature where extensive damage to the trailing edge existed [24,25,26]. Interestingly, there is no significant large-scale separation in the notch cavity of the turbine vanes in this study. However, the notch of the vane causes fluids near the vane to leave the trailing edge prematurely, and the expansion of the flow is weakened. The still higher transient pressure fields near the pressure and suction surfaces converge at the trailing edge, creating a localized high-pressure region. The deeper the notch depth, the higher the transient pressure at the trailing edge, which is evident in the current curved notch. The region in the notch cavity has the highest transient pressure where the notch depth is maximum at mid-span. In the spanwise direction from the midspan to the end wall, the expansion degree of the fluid near the blade gradually recovers as the notch depth decreases. It results in a localized bi-directional radial pressure gradient across the span of the notch. This contrasts with the unidirectional radial pressure gradient (nearly monotonic down-pressure trend from the shroud to the hub) at the trailing edge of the undamaged blade, as indicated by the blue arrow in Figure 10a,b.

**Figure 11:**
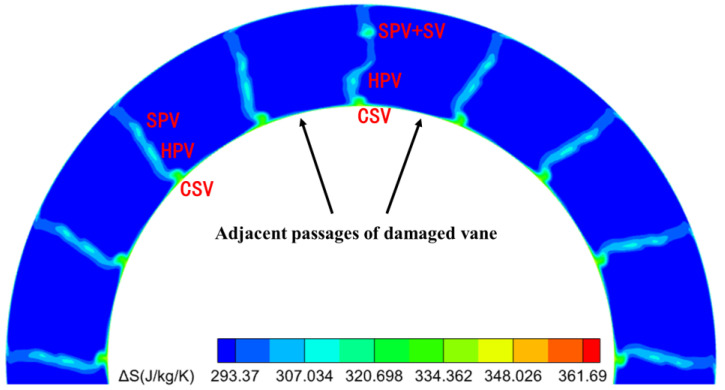
Contour of entropy production at the stator exit for Damage case.

The SPV flows from the upper part of the passage toward the mid-span in a normal path (red arrows in Figure 10b). When flowing to the notch edge, the SPV flips toward the shroud under the influence of the adverse pressure gradient generated by the localized high-pressure region of the notch. When it moves to the upper edge of the notch, it is again subjected to the adverse pressure gradient and flips back, forming a secondary vortex that rotates clockwise. Meanwhile, this secondary vortex induces a separating vortex (SV) that rotates counterclockwise around the vortex core due to the shear interaction of the boundary layer on the blade surface above the SPV. Compared with the undamaged blade passage, the SPV and SV at the exit together form a higher entropy production region with more concentrated and larger losses in the upper part of the span. This can be visualized in a contour plot of entropy [31,45,46] at the stator row exit (Figure 11). The entropy production ΔS is defined as:

(12) $$\Delta S=C_{p}\ln\frac{T}{T_{ref}}-R_{g}\ln\frac{P}{P_{ref}}.$$

Various types of viscous losses (passage vortex/corner separation, etc.), are shed/mixed/developed at the trailing edge and together they form the wake at the exit of the stator row. Entropy is also generally used to track or characterize the wake, so the contours of entropy in Figure 11 represent the wake distribution at the outlet of the stator vane. Total pressure or velocity characterization of the wake has also been considered in quantifying the wake strength. However, the total pressure loss can be used as a proper characterization for aerodynamic loss only in a steady flow, and the entropy wake is more accurate in non-stationary calculations [47]. In addition, since the velocity wake will include the velocity change in the mainstream, it does not simply characterize the velocity deficit of the wake. Therefore, entropy is finally chosen as the main means of tracing the wake in this study.

Damage to the vane trailing edge created a radial pressure gradient in both directions (Figure 10b). The pressure gradient from the center of the notch to the shroud induced the SPV to flip toward the shroud. Meanwhile, the boundary layer was induced to generate the SV, creating a more concentrated region of high entropy production. In combination with Figure 10 and Figure 11, it can also be observed that the pressure gradient from the center of the notch to the hub then drives the hub passage vortex (HPV) to travel in the radial direction toward the hub. As the flow reaches the lower edge of the notch, there is no adverse pressure gradient as there is at the upper edge. The HPV continues to flow obliquely downward out of the stator passage under the action of the favorable pressure gradient. More, the radial movement of the HPVs also causes the CSVs to be squeezed. The CSVs are closer to the hub, and their impact area is significantly reduced. The radial movement of the multiple vortices described above ultimately causes the wake profile of the damaged vanes to be significantly different from the other undamaged vanes, which can lead to an uneven distribution of the stator exit wake in the circumferential direction.

**Figure 12:**
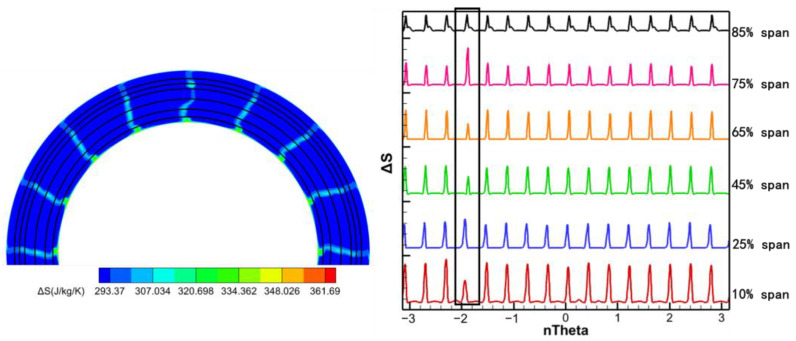
The circumferential distribution of entropy production at 10%, 25%, 45%, 65%, 75%, and 85% spans.

Extracting wake profiles at specific radial locations helps to deepen the understanding of the circumferential distribution of wake disturbances at stator exit. Figure 12 illustrates the circumferential distribution of entropy production at 10%, 25%, 45%, 65%, 75%, and 85% spans. To compare the entropy production distributions for all spans in a single figure, the entropy production curves for different spans are shifted along the vertical axis, and the values of the vertical axis in Figure 12 do not represent the true entropy production for different spans. The damaged vane wake exhibits three main features compared to the undamaged vane wake: (1) the wake strength is essentially the same at 85% span; (2) the aerodynamic loss is higher, and the wake strength increases at 25% and 75% spans; and (3) the wake strength weakens at 10%, 45% and 65% spans. The radial redistribution of the different secondary vortices at the exit of the damaged vanes leads to a different entropy production in these spans than in the undamaged vanes. Apparently, this is one of the reasons for the generation of low-order harmonic components at the stator row exit. Similarly, the localized high static pressure region at the trailing edge of the damaged vane may also be one of the sources of the low-order harmonic components in the flow field. 

The association between wake and entropy production has been described above, and either the circumferential uniform or non-uniform pressure distribution is regarded as a potential field perturbation by the downstream rotor blades. The aerodynamic excitation of rotor blades in the current subsonic turbine stage mainly comes from the stator row potential field disturbance and wake disturbance. The unsteady flow characteristics at the trailing edge of the damaged vane are dominated by the localized high static pressure region and the radial redistribution of the secondary vortices. They will lead to the non-uniform circumferential distribution of transient pressure and entropy. Analyzing the harmonic components of the potential field and wake at the stator row exit is an important procedure to reveal the LEO excitation mechanism.

**Figure 13:**
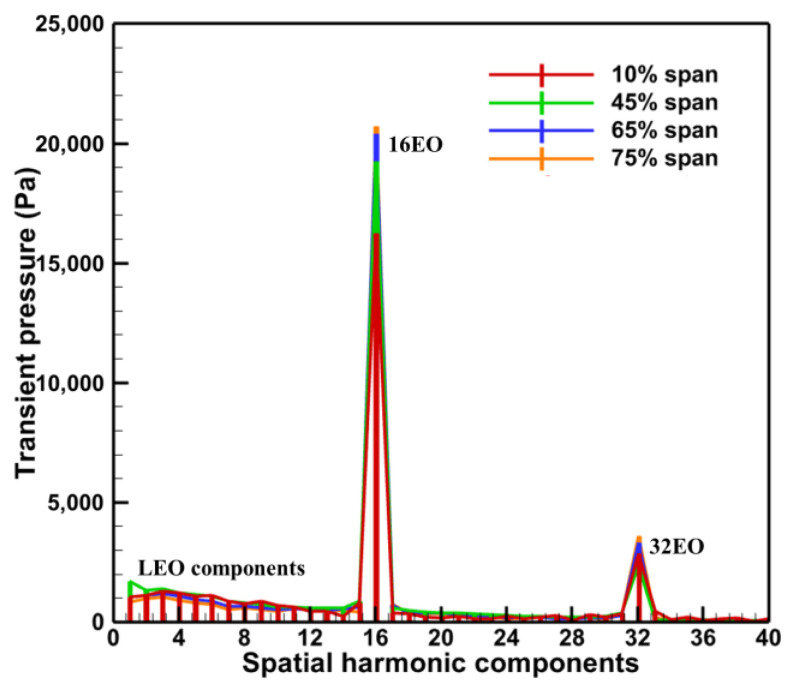
Spatial DFT of transient pressure distribution at the stator exit.

**Figure 14:**
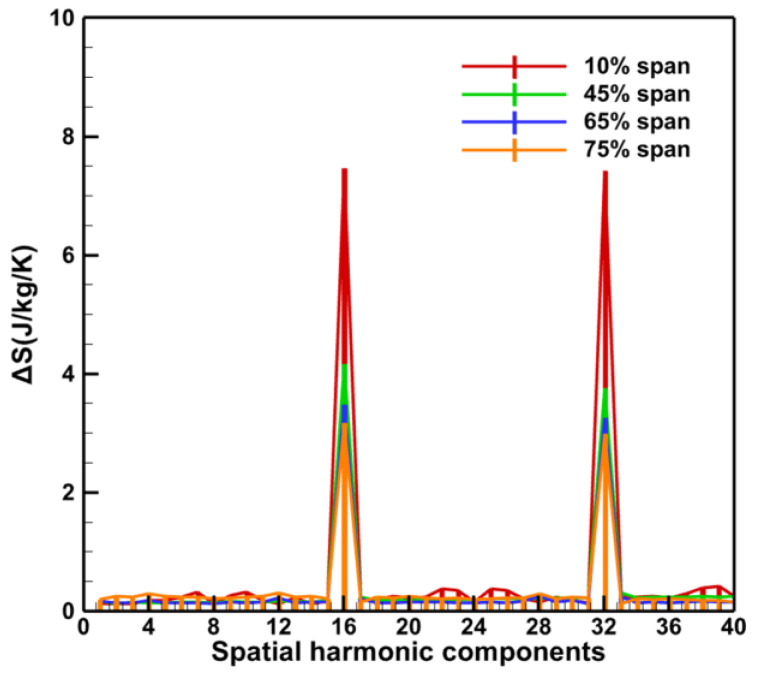
Spatial DFT of entropy production distribution at the stator exit.

The spatial DFT of entropy and transient pressure distribution at the stator row exit can be used to analyze the spatial harmonic components of the exit wake and potential field to determine the disturbance sources (wake and potential field) in the stator row with damaged vane. According to the spans that may have low-order harmonic components in Figure 12, spatial DFT was performed on the entropy and transient pressure contours for 10%, 45%, 65%, and 75% spans at the stator row exit (Figure 13 and Figure 14).

In Figure 13 and Figure 14, the spatial harmonic components of both the Baseline case and the Damage case are dominated by 16 and its multiples (32) related to the vane counts, but there is a significant low-order harmonic component in the Damage case. Moreover, it is important to note that only one damaged vane exists in the current stator row. However, both the wake and the potential field have a family of low-order harmonics. The 2nd and 3rd orders with higher amplitudes may be related to the influence of the damaged vanes in the adjacent passages, and the other lower order harmonics are present as multiples of the 1st–3rd orders. 

**Figure 15:**
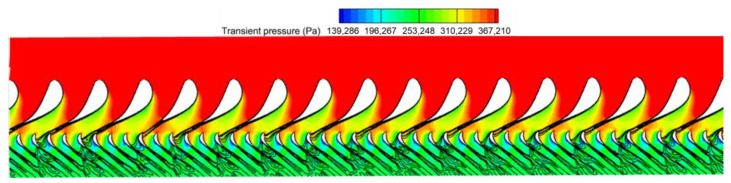
Schematic diagram of S1 flow field at 50% span at 3EO crossing.

A damaged vane produces transient pressure and entropy with non-uniform circumferential distribution, which gives rise to the spatial DFT spectral distribution with low-order harmonics at the stator row exit. These low-order harmonics propagate downstream and eventually act on the rotor blades and become the main source of unsteady pressures on the rotor blade. Figure 15 shows a schematic diagram of the S1 flow field at 50% span at 3EO crossing superposing the contours of the entropy and transient static pressure. The black entropy contours are used to trace the wake, while the background cloud of transient static pressure characterizes the potential field.

The unsteady effect of stator row disturbances on the rotor row is obvious. With each rotation of the rotor blades through the stator row potential field and wake, the circumferentially unevenly distributed velocity field/pressure field in the absolute reference system is regarded as a distorted incoming flow in the rotor reference system. The periodic relative rotation of the two blade rows causes periodic variations in the transient pressure on the rotor blade surfaces (Figure 8 and Figure 9). As mentioned above, the time domain curve of transient pressure on rotor blades in the Baseline case (Figure 8) shows 16 complete sinusoidal periods (corresponding to the vane counts) with more consistent amplitudes of peaks and troughs. However, in the Damage case (Figure 9), the time domain curve of transient pressure contains only 13 similar sinusoidal periods, and the other 3 sinusoidal periods have different amplitudes of peaks and troughs. Combined with the analysis of the flow field at the stator exit (Figure 10, Figure 11, Figure 12, Figure 13 and Figure 14), the damaged vane causes a variation in the perturbation strength of the potential field and wake in the two passages adjacent to the pressure and suction sides.

More, the low-order harmonics of the stator row wake and potential field perturbations may not contribute to the LEO resonance response of the rotor blades to the same extent. Determining the contributions of the wake flow and potential field to the aerodynamic excitation of the blade surface can help to provide insight into the LEO excitation mechanism induced by a single damaged blade. If damage occurs at other spans than the midspan on the blade in the engineering, this underlying mechanism may be used to identify the resonance risk in advance of blade failure.

**Figure 16:**
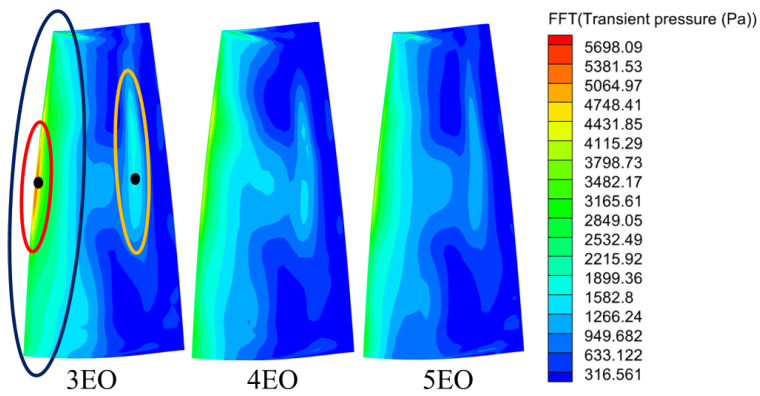
The amplitude of 3–5 EO excitation on rotor blades.

Figure 16 illustrates the amplitude distribution of the aerodynamic excitation for 3–5 EO on the rotor blades. The results show that the distribution patterns of 3–5 EO aerodynamic excitation on the suction surface of the rotor blades are almost the same, and only the amplitude of each region is somewhat different. This suggests that the interaction mechanisms of different orders aerodynamic excitation may be similar, and it is feasible to analyze 3EO aerodynamic excitation as an example.

The regions affected by different EOs aerodynamic excitation are shown as the leading edge of the suction surface on the rotor blade (blue ellipse) and the mid-rear part of the blade (orange ellipse). The blade leading edge exhibited the LEO aerodynamic excitation at the whole span, with the highest magnitude of aerodynamic excitation at the mid-span of the blade (red ellipse). This is extremely similar to the radial distribution of the stator wake and potential field. It can be preliminarily presumed that the perturbations at each span at the stator exit propagate along the axial direction and will subsequently impinge on the leading edge of the rotor. The localized high-pressure region of the potential field in mid-span may contribute to the highest excitation amplitude in the mid-span of the rotor blades. In addition, another region with high amplitude exists at 40–60% axial chord of the rotor blade. In combination with Figure 15, the propagation of stator wake disturbance and potential field disturbance in the rotor passages may also be one of the aerodynamic sources of localized LEO excitation on the rotor blades.

**Figure 17:**
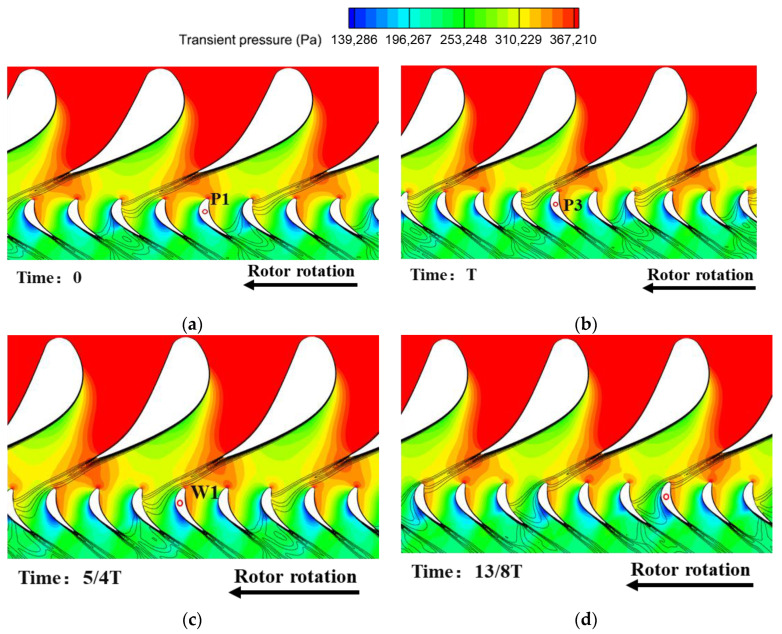

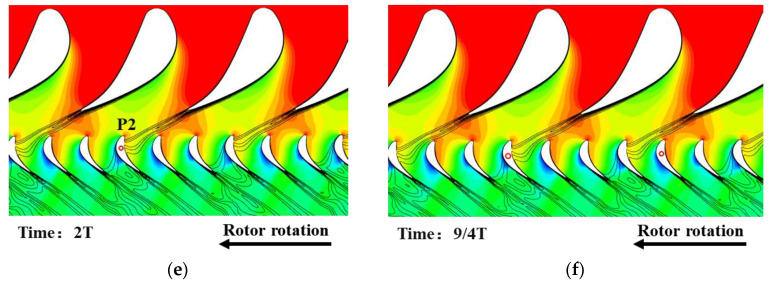
S1 flow field of turbine stage at multiple time steps. (**a**) Time = 0; (**b**) Time = T; (**c**) Time = 5/4T; (**d**) Time = 13/8T; (**e**) Time = 2T; (**f**) Time = 9/4T.

**Figure 18:**
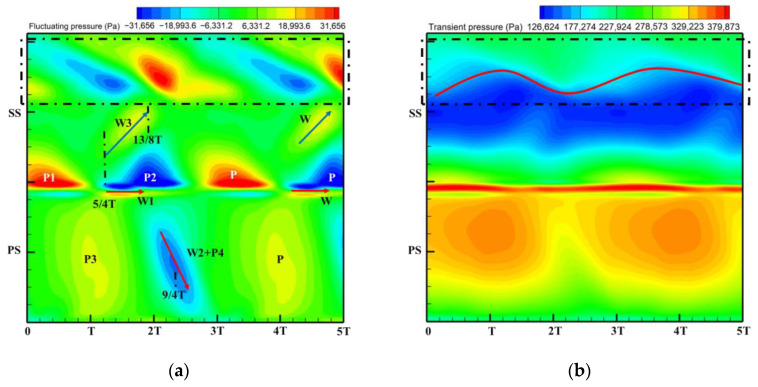
Time–space diagram of rotor blade for Baseline case: (**a**) Fluctuating pressure; (**b**) Transient pressure.

The excitation mechanism of the stator row wake and potential field disturbances on the rotor blade surface are discussed in Figure 17 and Figure 18 to explain the sources of the high amplitude regions in the excitation distribution on the blade surface in Figure 16. The fluctuating pressure in this paper is obtained by subtracting the time-averaged pressure of one rotor revolution from the transient pressure. The horizontal coordinates in the time–space diagram (Figure 18) represent the time scale (iteration steps), which is characterized by the time step range comparable to the time required for the rotor to rotate through five rotor passages. Each rotation of the rotor blade through one rotor passage is defined as one time period T, so the horizontal coordinate represents a time step ranging from 0 to 5T. In addition, the time scale for the rotor to rotate through six rotor passages is similar to the time it takes for the rotor to rotate through two stator passages. The vertical coordinates in the time–space diagrams then represent the transient pressure distributions at 50% span for the pressure surface (PS) and suction surface (SS) of the monitored rotor blades (blades marked by red circles in Figure 17). The corresponding positions of the PS and the SS are labeled in the figure. Each high- or low-pressure region has been noted with a letter, where P means potential field excitation and W represents wake excitation.

When Time = 0 (Figure 17a), the monitored rotor blade approaches the stator trailing edge and it is going to be impacted by the high-pressure region of the potential field of the stator trailing edge. Then during the time from Time = 0 to Time = T (Figure 17b), the rotor makes a complete rotation through the high-pressure region of the tailing potential field. This is a process of gradual approaching and leaving of the high-pressure potential field for the rotor leading edge, which is reflected in the high amplitude region P1 for the time–space diagram (Figure 18a). Meanwhile, the blade pressure surface is impacted by the high amplitude of the stator potential field at the T moment that forms the P3 region.

As the time comes to Time = 5/4T (Figure 17c), the upstream stator vane wake gradually approaches the leading edge of the monitored blade. As the rotor blade continues to rotate, the upstream stator blade wake will be cut into two parts by the rotor blade. One part of the wake directly impinges on the pressure leading edge of the rotor blade and then propagates downstream along the pressure side passage of the monitored blade. When the rotor continues to rotate, this portion will continue to act on the rotor pressure surface and form the W2 region.

Another portion of the wake enters the suction side passage of the monitored blade during the time period from Time = 5/4T to Time = 13/8T (Figure 17d). Due to the effect of the crosswise pressure difference between the pressure and suction surfaces in this passage, the wake propagates downward from the leading edge along the blade profile, and this process forms the W3. Meanwhile, in the time period from Time = 13/8T to Time = 2T (Figure 17e), the leading edge of the monitored rotor blade is located downstream of the main flow passage of the stator row, and the low-amplitude region of the potential field attacks the leading edge of the rotor to form the P2 region. Finally, at Time = 2T and Time = 9/4T (Figure 17f), the low-amplitude region of the potential field will also directly enter the rotor passage to impact the blade pressure surface to form the P4 region.

In addition, near the trailing edge of the suction surface, the time–space diagram of the fluctuating pressure shows alternating high and low amplitudes. By analyzing the transient pressure time–space diagram (Figure 18b), these alternating pressures are caused by the change in the position of the shock wave on the suction surface. Although there is a subsonic flow between the stator and rotor rows, there is no shock wave interference. However, the rear part of the rotor passage is transonic flow and there is a shock wave (obvious pressure spike demarcation—red solid line). Since the position and intensity of the shock wave depend on the Mach number (Ma) prior to the shock wave, the potential field and wake acting on the suction surface of the rotor (especially the w3 of the velocity deficit) will inevitably result in a variation of Ma. This ultimately results in the periodic shift in the position of the shock wave on the blade surface observed in Figure 18b.

**Figure 19:**
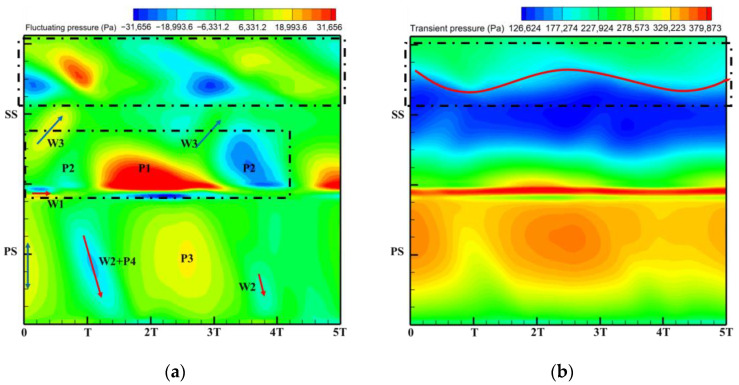
Time–space diagram of rotor blade for Damage case: (**a**) Fluctuating pressure; (**b**) Transient pressure.

Figure 19 shows the time–space diagrams of fluctuating and transient pressures at the blade surface of the monitored rotor at 50% span for the Damage case. By comparing with the Baseline case time–space diagrams, the source of the high amplitude region of the low-order excitation on the blade surface in Figure 16 is analyzed.

First, the localized high pressure in the notch cavity of the damaged stator (Figure 10b) leads to an increase in the intensity of the level of the potential field acting on the leading edge of the rotor, which is reflected in the enhancement of the high amplitude P1 dominated by the potential field. In addition, the radial redistribution of the secondary vortices due to the radial pressure gradient (Figure 11) leads to a significant weakening of the wake intensity in the midspan. This corresponds to a diminished W3 in the front-middle of the suction surface. It is confirmed by the weakening of W2 at the pressure surface of the monitored rotor blades. The weakening of leading edge P2 may be related to the expansion of the adjacent passage of the damaged vane. In addition, changes in the strength of the wake and potential field will also significantly change the intensity and location of the shock wave at the suction surface (Figure 19b). These flow field variations described above ultimately become the source of the low-order excitations in the blade surface in Figure 16.

Thus far, the low-order harmonic excitation at the exit due to the single damaged vane acts mainly at the leading edge of the rotor blade and the mid-chord area of the suction surface, but the interaction mechanism is not the same at the two regions. The high amplitude of low-order excitation at the leading edge presents the combined effect of multiple perturbations dominated by the potential field. The high amplitude of low-order excitation at the middle chord is closely related to the variation of the intensity and position of the shock wave due to the propagation of the stator row perturbation in the rotor row.

**Figure 20:**
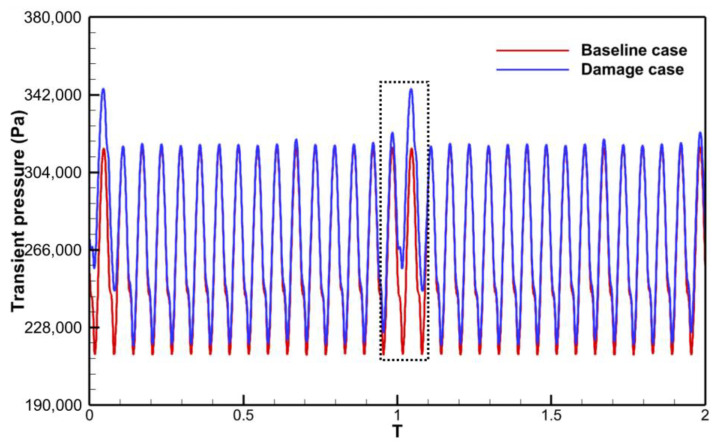
Time histories of transient pressures at leading edge points of the suction surface.

As known from previous work [31], the strength of the LEO aerodynamic excitation is determined by the variation level in the average value of the transient pressure. The inconsistency in the interaction mechanism between these two regions can also be identified by the time domain diagrams of the transient pressure curves. The transient pressure was monitored by picking up a point (Figure 16) in the high amplitude region of the leading edge of the rotor blade and the mid-chord of the suction surface, respectively. The transient pressure at the leading edge point (Figure 20) exhibits increased peaks and valleys shape as the point passes through the damaged vane passage (Black dashed box). The increase in the average transient pressure is the source of the LEO excitation with high amplitude. This also coincides with the localized high-pressure region of the trailing edge notch of the damaged vane (Figure 10b) and the P1 region in Figure 19a. It also verifies that the low-order aerodynamic excitation at the rotor leading edge is mainly caused by multiple perturbations dominated by the potential field impinging on the rotor leading edge.

**Figure 21:**
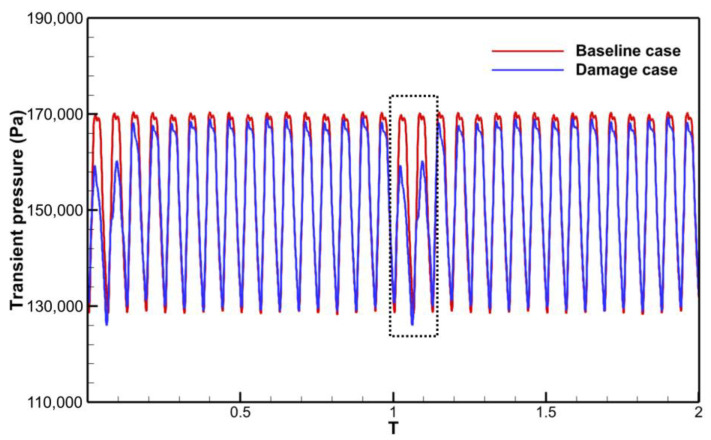
Time histories of transient pressures at the mid-rear point of the suction surface.

In contrast, the transient pressure of the monitoring point at the mid-rear of the suction surface (Figure 21) exhibits a reduction in the peaks and troughs of the transient pressure as it passes through the passage of the damaged vane (Black dashed box). The average value reduction of transient pressure is responsible for the LEO excitation with high amplitude at this region. This just confirms that the stator row disturbance propagating through the rotor row causes a weakening of the shock wave at the rear of the blade as mentioned above. In short, on the one hand, since present engine designs usually pursue smaller axial spacing, this may worsen the resonance risk caused by sudden vane damage. On the other hand, the inconsistency in the excitation mechanisms at different regions of the rotor blade may make the selection of aerodynamic measures to control resonance response more cautious in the future. For example, the adjustment of aerodynamic measures (solidity, axial spacing, etc.), for the leading edge, which is dominated by the potential field excitation, may not necessarily have a positive effect on the excitation level at the rear of the rotor blade. This needs to be focused on in future studies.

### 6.4. Aerodynamic Performance

Understanding the aerodynamic performance is also important to ensure high-efficiency operations and detect faults in time during turbine stage operation. The aerodynamic efficiencies of the different cases for the 3EO crossing are shown in Table 4.

Compared to the Baseline case, the change in aerodynamic efficiency after vane damage (Damage case) is very small, decreasing by only 0.018%. Such a performance fluctuation may be ignored in engineering. Unfortunately, a single damaged vane greatly increases the LEO resonance risk. Then, if trailing edge damage occurs to one vane at a stator row during engine operation, this failure may not be easily detected due to the insignificant change in aerodynamic efficiency. However, the vibration risk at the high level that it brings can greatly endanger the safe operation of the engine.

## 7. Conclusions

In this paper, a full annulus unsteady aeroelastic simulation of a single-stage turbine is carried out using an in-house CFD code. The effect of a single damaged vane of the stator row on the LEO forced response of the rotor blade is investigated. Firstly, this study finds that the damaged vane introduces multiple LEO harmonic components with high amplitude, which will substantially increase the LEO resonance risk of the rotor blades. Then the generation and interaction mechanisms of LEO aerodynamic excitation are clarified in terms of detailed flow mechanisms. Finally, the danger level of a single damaged vane is comprehensively evaluated in conjunction with the aerodynamic performance changes. The main conclusions are as follows:

1. A single damaged vane introduces not only 1EO excitation corresponding to damaged vane count but also a family of LEO aerodynamic excitations ranging from 2-10EO. For the single-stage turbine studied, the M1/3ND resonance response of the rotor blade excited by 3EO is 2.01 times higher than that of the M2/16ND resonance response excited by VPF. The resonance response of the corresponding ND excited by 4EO and 5EO with lower amplitude also reaches 1.797 and 1.804 times the resonance response excited by VPF, respectively, and the risk of blade fatigue failure of the blade is greatly increased;
2. The wake and potential field are the main disturbances for current turbine rotor blades. The localized high pressure in the notch cavity and the radial redistribution of the secondary vortex at the stator exit caused by the damaged vane are the main reasons for the generation of low-order harmonic components in the stator exit flow field. With the axial propagation of wake and potential field disturbances through the blade rows, the rotor blade surface is subjected to different intensities of LEO aerodynamic excitation. Advanced engine designs generally aim for smaller axial spacing, which may make the hazards caused by sudden blade damage more severe in the future;
3. This paper analyzes the excitation mechanisms of the whole rotor blade affected by the damaged vane. The low-order harmonics at the stator outlet due to a single damaged vane mainly act at the leading edge of the rotor blade and the mid-chord of the suction surface, and the excitation mechanisms in the two regions are not the same. The high excitation region at the leading edge exhibits the combined effect of multiple disturbances dominated by the potential field. The high excitation region at the mid-chord, on the other hand, is closely related to the weakening intensity of the shock wave at the blade surface. The increase and decrease in the average transient pressure are the main reasons for the higher LEO amplitude in the two high excitation regions, respectively. This will require a more careful selection of the various aerodynamic measures to control resonance response in the future.

This study comprehensively reveals, for the first time, the underlying mechanism of LEO resonance response induced by the damaged vane of the stator row from the perturbation source, aerodynamic excitation, to resonance response. This will help engineers consider more appropriate aerodynamic measures to control the LEO resonance response in blade design and provide them with theoretical support to develop more proper structural designs to optimize the vibration behavior of rotor blades in the future. However, only one damaged vane case was investigated in the current study. In engineering applications, the number of stator vanes experiencing damage failure is often random. There might be multiple vane damages with randomly distributed circumferential positions, and the interaction of unsteady perturbations between blade rows will be more complicated. It is also important to find out how to counteract these negative effects in future actual operations. The above will be the focus of attention in future studies.

## Figures and Tables

**Table 1:** Geometry parameters and operating conditions.

Geometry Parameter		
Parameter	Stator Row	Rotor Row
Airfoil count	16	47
Axial chord (midspan) (mm)	97	33
Airfoil height (mm)	75	74
Aspect ratio (exit height/chord)	0.77	2.24
Rotor tip clearance (mm)	/	1
Operating condition		
Total pressure at stator inlet	510,000 Pa	
Total temperature at stator inlet	1480 K	
Static pressure at rotor exit	237,000 Pa	
Design rotation speed $\;n_{design}$	15,400 rpm	

**Table 2:** The simulation results of different CFD codes.

Parameter	Aerodynamic Efficiency	Expansion Ratio
HGAE	0.8954	1.7616
NUMECA	0.8988	1.7587
Error	0.378%	0.165%

**Table 3:** Comparison of $X_{max,nor}$ for different cases.

Case (NDs)	EO Excitation	Resonant Speed	$X_{max,nor}$
Baseline case (16 NDs)	16 EOs	$68\%\;n_{design}$	1
Damage case (3 NDs)	3 EOs	$96\%\;n_{design}$	2.009
Damage case (4 NDs)	4 EOs	$73\%\;n_{design}$	1.797
Damage case (5 NDs)	5 EOs	$60\%\;n_{design}$	1.804

**Table 4:** Comparison of aerodynamic performance.

Case	Mass Flow (kg/s)	Expansion Ratio	Stage Aerodynamic Efficiency
Baseline case	26.0776	1.755	89.589%
Damage case	26.1333	1.753	89.563%
Error	0.213%	−0.119%	−0.018%

## Data Availability

Data are contained within the article.
